# Supplementary material for: Neutrophil azurophilic granule glycoproteins are distinctively decorated by atypical pauci- and phosphomannose glycans
Source: Commun Biol. 2021 Aug 26;4:1012. doi: 10.1038/s42003-021-02555-7 (PMC8390755; doi:10.1038/s42003-021-02555-7)
Supplement: Supplementary file 2 — Supplementary Information [file 42003_2021_2555_MOESM2_ESM.pdf]

SUPPLEMENTARY FIGURES

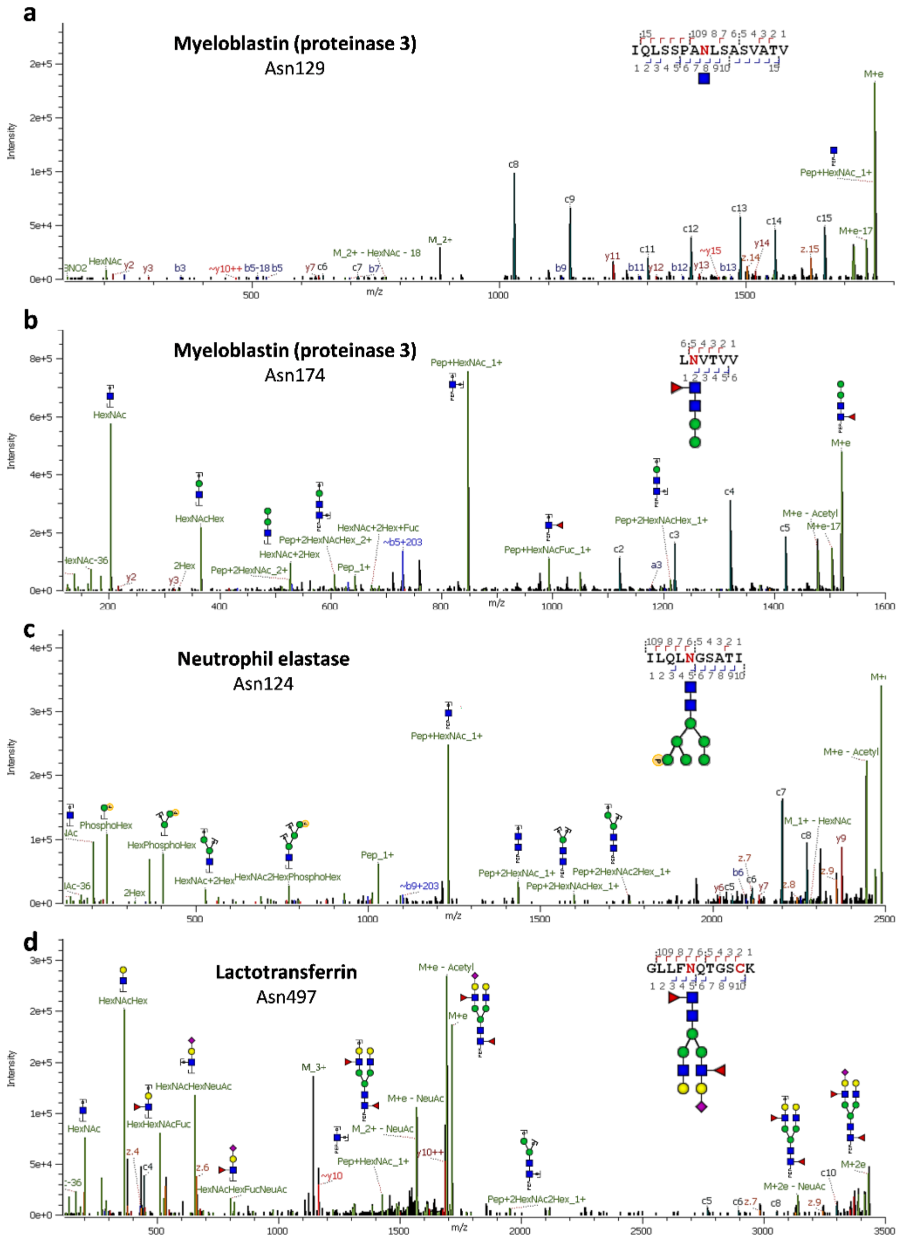

Supplementary Figure 1: Neutrophil glycopeptide MS/MS spectra as generated by EThcD. a) Localization of the GlcNAc to myeloblastin Asn129, most distinguishable by clear C8 and C9 fragments carrying the monosaccharide residue. **b**) Localization of paucimannose glycosylation to myeloblastin Asn174, indicated by glycosylated C2 and C3 fragments. **c**) Behaviour of phosphomannosylated neutrophil elastase glycopeptides in EThcD. **d**) Lactoferrin glycopeptide showing $HexNAc_1Hex_1Fuc_1NeuAc_1$ oxonium ions (*m/z* 803.29), suggesting sialyl-Lewis A/X to be at least partially present for this glycopeptide composition. Note that the fragments above may have originated from a mixture of isomeric structures, wherein alternative fragmentation pathways could lead to observed ions like $HexNAc_1Hex_1NeuAc_1$ (*m/z* 657.23) and $HexNAc_1Hex_1dHex_1$ (*m/z* 512.20).

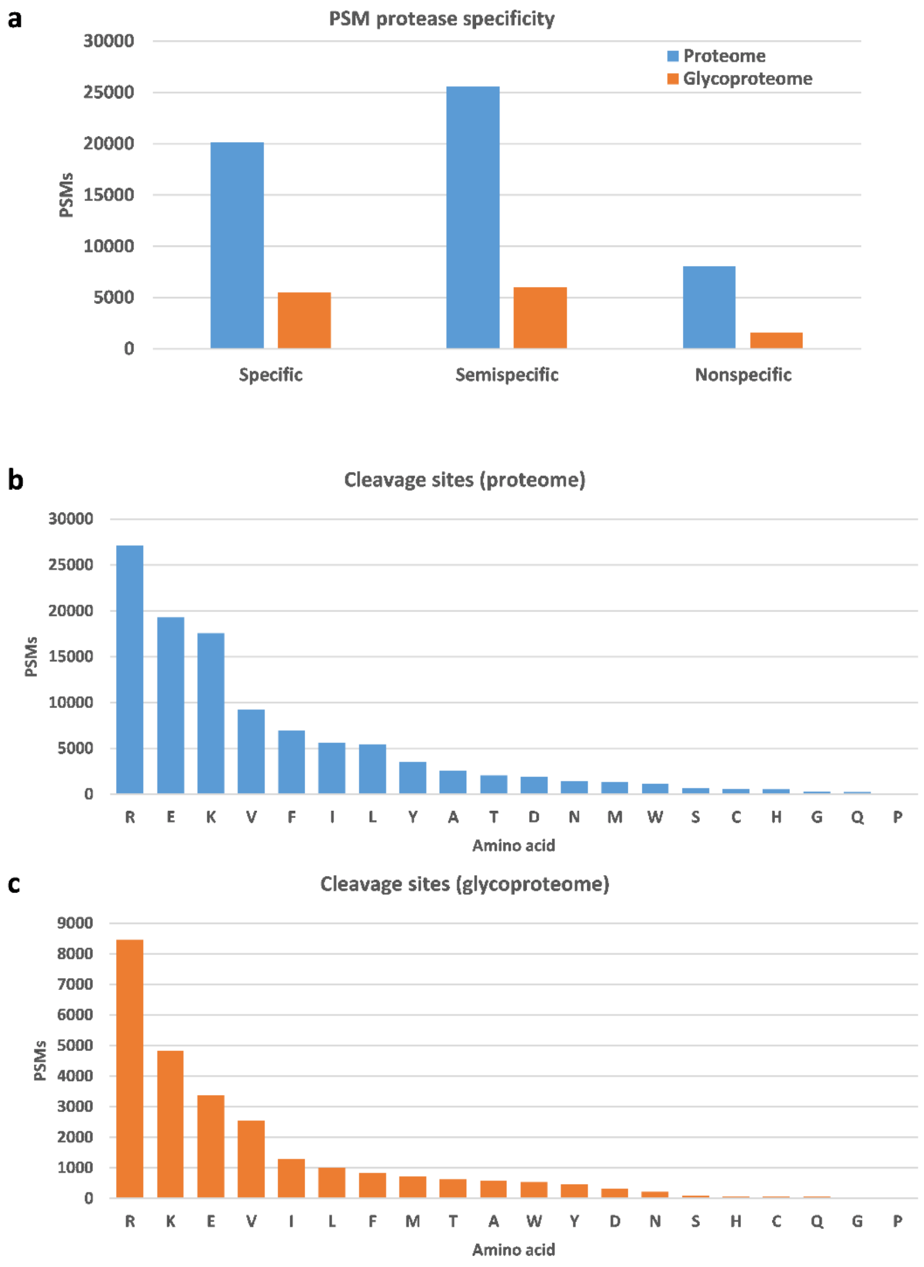

Supplementary Figure 2: **Overview of cleavage specificity**. **a**) Total count of PSMs that are either specific (both cleavage sites for a given peptide being as expected for the included protases, namely at Arg, Lys, Glu or Asp), semi-specific (only one of the cleavages being as expected) and non-specific (neither of the cleavage sites being as expected). Further distinction was made for individual amino acids involved in the cleavage for either **b**) the whole neutrophil proteome or **c**) only the glycoproteome. As can be seen, while cleavage does occur at the expected Arg, Lys, Glu (and Asp) residues, frequent cleavage also occurs at Val, Phe, Leu, Ile, and other residues.

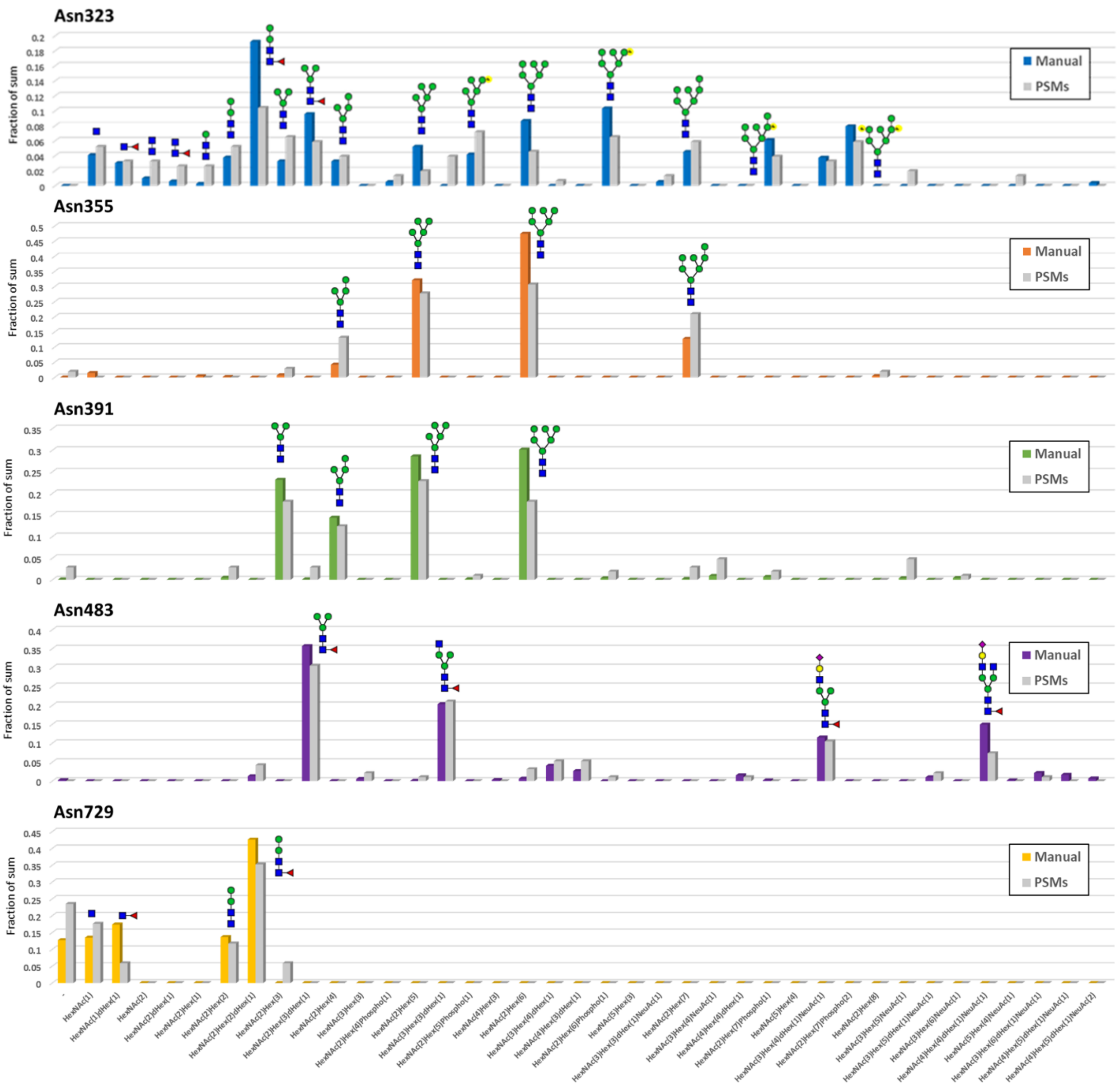

Supplementary Figure 3: Site-specific myeloperoxidase glycosylation compared between manual area integration *via* Skyline (manual, MS^1-based) and the counting of peptide spectral matches resulting from glycoproteomics searches in Byonic (PSMs, MS^2-based). While not exactly identical, the methods are highly congruent in the glycan compositions identified and approach each other in terms of relative quantification as well for each of the distinct myeloperoxidase *N*-glycosylation sites.

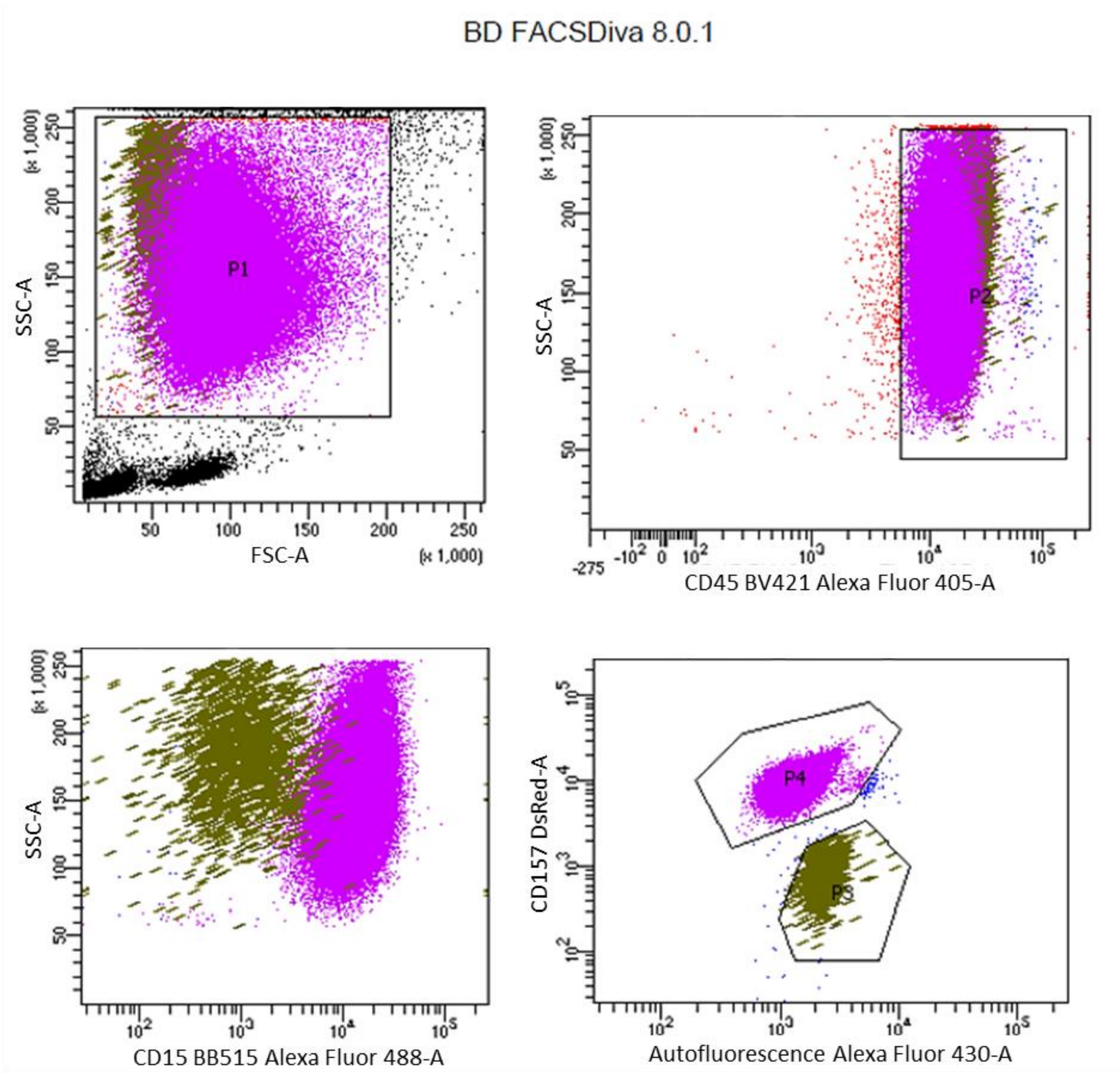

Population	#Events	%Parent	%Total
All Events	117,601	####	100.0
P1	100,203	85.2	85.2
P2	99,506	99.3	84.6
P3	1,636	1.6	1.4
P4	97,753	98.2	83.1

Supplementary Figure 4: **Overview of fluorescence-activated cell sorting (FACS) strategy**. After isolation from buffy coats by density gradient centrifugation, human neutrophils (P4) were selected by FACS as having high side scatter (SSC-A), intermediate CD45 expression, high CD15 and CD157 expression with dim autofluorescence in the AF430 channel.
