# Supplementary material for: Neutrophil azurophilic granule glycoproteins are distinctively decorated by atypical pauci- and phosphomannose glycans
Source: Commun Biol. 2021 Aug 26;4:1012. doi: 10.1038/s42003-021-02555-7 (PMC8390755; doi:10.1038/s42003-021-02555-7)
Supplement: Supplementary file 3 — Description of Supplementary Files [file 42003_2021_2555_MOESM3_ESM.pdf]

Description of Additional Supplementary files

File name: Supplementary Data 1

Description: Glycan compositions.

File name: Supplementary Data 2

Description: Shotgun MS results with specific search.

File name: Supplementary Data 3

Description: Shotgun MS results with nonspecific search.

File name: Supplementary Data 4

Description: Glycoproteomics results (HCD and EThcD).

File name: Supplementary Data 5

Description: Glycoproteomics results (EThcD).
