# Supplementary material for: Neutrophil azurophilic granule glycoproteins are distinctively decorated by atypical pauci- and phosphomannose glycans
Source: Commun Biol. 2021 Aug 26;4:1012. doi: 10.1038/s42003-021-02555-7 (PMC8390755; doi:10.1038/s42003-021-02555-7)
Supplement: Supplementary file 9 — Reporting Summary [file 42003_2021_2555_MOESM9_ESM.pdf]

nature research

Corresponding author(s): Karli R. Reiding
Albert J.R. Heck

Last updated by author(s): May 31, 2021

Reporting Summary

Nature Research wishes to improve the reproducibility of the work that we publish. This form provides structure for consistency and transparency in reporting. For further information on Nature Research policies, see our Editorial Policies and the Editorial Policy Checklist.

Statistics

For all statistical analyses, confirm that the following items are present in the figure legend, table legend, main text, or Methods section.

n/a	Confirmed	
☐	☒	The exact sample size (*n*) for each experimental group/condition, given as a discrete number and unit of measurement
☐	☒	A statement on whether measurements were taken from distinct samples or whether the same sample was measured repeatedly
☒	☐	The statistical test(s) used AND whether they are one- or two-sided<br>*Only common tests should be described solely by name; describe more complex techniques in the Methods section.*
☒	☐	A description of all covariates tested
☒	☐	A description of any assumptions or corrections, such as tests of normality and adjustment for multiple comparisons
☒	☐	A full description of the statistical parameters including central tendency (e.g. means) or other basic estimates (e.g. regression coefficient) AND variation (e.g. standard deviation) or associated estimates of uncertainty (e.g. confidence intervals)
☒	☐	For null hypothesis testing, the test statistic (e.g. *F*, *t*, *r*) with confidence intervals, effect sizes, degrees of freedom and *P* value noted<br>*Give P values as exact values whenever suitable.*
☒	☐	For Bayesian analysis, information on the choice of priors and Markov chain Monte Carlo settings
☒	☐	For hierarchical and complex designs, identification of the appropriate level for tests and full reporting of outcomes
☒	☐	Estimates of effect sizes (e.g. Cohen's *d*, Pearson's *r*), indicating how they were calculated

Our web collection on statistics for biologists contains articles on many of the points above.

Software and code

Policy information about availability of computer code

Data collection	Data was collected using a Q Exactive HF mass spectrometer (Thermo Fisher Scientific, Bremen, Germany) and an Orbitrap Fusion Lumos mass spectrometer (Thermo Fisher Scientific, Bremen, Germany) both with accompanying software from Thermo Fischer Scientific.
Data analysis	Proteome Discoverer (version 2.3, Thermo Scientific) was used to analyse the LC-MS2 raw files from the shotgun experiment. The raw files acquired from the glycoproteomics experiments were searched with Byonic (version 3.7.13, Protein Metrics Inc.).

For manuscripts utilizing custom algorithms or software that are central to the research but not yet described in published literature, software must be made available to editors and reviewers. We strongly encourage code deposition in a community repository (e.g. GitHub). See the Nature Research guidelines for submitting code & software for further information.

Data

Policy information about availability of data

All manuscripts must include a data availability statement. This statement should provide the following information, where applicable:

- Accession codes, unique identifiers, or web links for publicly available datasets
- A list of figures that have associated raw data
- A description of any restrictions on data availability

All raw data and processed files generated throughout the study are available through the MassIVE repository accessible via ftp://MSV000086020@massive.ucsd.edu using the password "NPGP_01".

Field-specific reporting

Please select the one below that is the best fit for your research. If you are not sure, read the appropriate sections before making your selection.

☒ Life sciences ☐ Behavioural & social sciences ☐ Ecological, evolutionary & environmental sciences

For a reference copy of the document with all sections, see nature.com/documents/nr-reporting-summary-flat.pdf

Life sciences study design

All studies must disclose on these points even when the disclosure is negative.

Sample size	A single pool of neutrophils was studied by nine mass spectrometric runs across three sample preparation conditions.
Data exclusions	For the proteomics data the following curations were performed: The searches were performed using a precursor mass tolerance of 10 ppm and a fragment mass tolerance of 0.05 Da followed by data filtering using Percolator, resulting in a 1% false discovery rate (FDR). For label-free quantification, the node called Minora feature detector was used with high PSM confidence, a minimum of 5 non-zero points in a chromatographic trace, a minimum number of 2 isotopes and maximum retention time difference of 0.2 min for isotope peaks. The consensus workflow in Proteome Discoverer was used to open the search results and enable retention time (RT) alignment with a maximum RT shift of 5 min and mass tolerance of 10 ppm in order to match the precursor between runs. Proteins were only reported when detected in at least two out of three replicate and covered by at least three unique peptides. For glycoproteomics data we included the following constraints: The precursor ion mass tolerance was set to 10 ppm and both HCD & EThcD fragment mass tolerance were set to 20 ppm.
Replication	Our observations were consistent across mass spectrometric measurement regimes, and the newly reported glycosylation features were replicated for many proteins within the study.
Randomization	No particular randomisation was performed, we report new observations rather than compare study populations.
Blinding	No blinding was performed, all data was analysed by defined methods and constraints, our reported observations are entirely data-driven.

Reporting for specific materials, systems and methods

We require information from authors about some types of materials, experimental systems and methods used in many studies. Here, indicate whether each material, system or method listed is relevant to your study. If you are not sure if a list item applies to your research, read the appropriate section before selecting a response.

Materials & experimental systems

n/a	Involved in the study	
☒	☐	Antibodies
☒	☐	Eukaryotic cell lines
☒	☐	Palaeontology and archaeology
☒	☐	Animals and other organisms
☒	☐	Human research participants
☒	☐	Clinical data
☒	☐	Dual use research of concern

Methods

n/a	Involved in the study	
☒	☐	ChIP-seq
☒	☐	Flow cytometry
☒	☐	MRI-based neuroimaging
